# The fat regulator neuropeptide Y and caloric restriction

**DOI:** 10.18632/aging.101338

**Published:** 2017-11-30

**Authors:** Seongjoon Park, Ryoichi Mori, Isao Shimokawa

**Affiliations:** Department of Pathology, Nagasaki University School of Medicine, Graduate School of Biomedical Sciences, Nagasaki City 852-8523, Japan

**Keywords:** neuropeptide Y, fat metabolism, caloric restriction

Fat provides an essential energy source in mammals when other supplies are an insufficient. Body fat tends to increase up to middle age, but decreases thereafter. The high levels of body fat in middle aged animals suggest that the balance between fat storage and oxidation, which is important to maintain metabolic homeostasis, is disturbed in this period. This can lead to increased visceral fat accumulation and metabolic disease, whereas abnormal fat loss can induce lipo-dystrophy. Lower body fat is a common phenotypic trait in animal models of longevity, including models of caloric restriction (CR). However, the obesity paradox suggests that increased fat content can also be protective, as mice with higher fat content live longer [1], suggesting that body weight or fat reduction is not definitively the best path to longevity.

Neuropeptide Y (NPY) is a key orexigenic neuropeptide that regulates adiposity by attenuating energy storage and utilization. Fasting or CR stimulates hypothalamic NPY expression, thereby increasing appetite. NPY is mainly expressed in the hypothalamus but is also expressed in peripheral tissues, such as white adipose tissue and bone cells, indicating that it not only plays a role in maintaining energy homeostasis but also in regulating physiological functions. However, its physiological functions in peripheral tissues have not been clearly determined. Recently, we showed that NPY is closely linked to lipid accumulation and mobilization, by regulating peroxisome proliferator-activated receptor gamma 2 and hormone sensitive lipase (HSL). Moreover, NPY deficiency ameliorates factors contributing to age-related imbalances in adipose tissue metabolism, such as increased inflam-mation, decreased *de novo* lipogenesis in visceral fat, and reduced thermogenic activity in subcutaneous fat [2]. However, modification of fat metabolism by NPY deficiency is sex-dependent. NPY deficiency does not affect adiposity in male mice and could be involved in gonadal hormones regulation. Further investigations are required to determine the sexual dimorphism of NPY in the regulation of fat metabolism.

Contrary to its beneficial function in positive energy balances, NPY also appears to be necessary in negative energy balances. In a previous study, we found that NPY deficiency disrupts CR-mediated lifespan exten-sion [3]. This finding provided the first demonstration that NPY plays an important role in the adaptation to negative energy balances. The effects of CR on the regulation of cancer and stress responses were attenuated by NPY deficiency, while physiological processes activated by CR, including inhibition of the insulin-like growth factor 1 and mammalian target of rapamycin (mTOR) pathways and the modulation of adipokine and corticosterone levels, were not affected by NPY deletion. To better understand how NPY deletion disrupts the lifespan-extending effect of CR, we monitored body weight, body fat percentage, and fat metabolism. Interestingly, NPY deficiency increased mortality in CR male mice in association with body weight reduction and fat loss in middle ages, by upregulating lipolysis/thermogenesis via marked activation of the adrenergic receptor beta 3-HSL signaling pathway, and this was inhibited by treatment with the lipolysis inhibitor, acipimox [4]. These results suggest that the control of lipid metabolic homeostasis by NPY is critical for the lifespan-extending effect of CR. The finding that CR modifies metabolic patterns characterized by increased fatty acid synthesis and oxidation in mice [5] prompted us to investigate the role of NPY in CR-regulated fat metabolism. We used next-generation sequencing (NGS) to discover that under CR, NPY controls β-adrenergic remodeling in subcutaneous fat. A master transcriptional regulator of fatty acid biosynthesis, sterol regulatory element–binding protein 1c (SERBP1c), which is mainly expressed in liver and white adipose tissue, is involved in modulating β-adrenergic stimulation and is required for CR-associated longevity [6]. However, Bruinstroop et al. showed that NPY administration did not affect SREBP1c expression in the liver [7]. Furthermore, SREBP1c expression was not modified by NPY deficiency under CR in our NGS analysis. Thus, NPY may not be involved in CR-mediated longevity effects regulated by SERBP1c.

Several longevity-related genes critical for the effects of CR, such as sirtuins, 5′-AMP-activated protein kinase catalytic subunit alpha-2 (aak-2), Forkhead box protein O (daf-16), beta-glucan synthesis-associated protein SKN1 (SKN1), and TOR have been identified by epistasis analysis in invertebrates, but mammalian studies have not demonstrated reproducibility. Inves-tigating longevity mechanisms through CR-specific energy metabolism might not only help clarify the correlation between fat metabolism and lifespan regulation, but also lead to the discovery of novel longevity pathways. Our findings clearly show that the maintenance of fat metabolic homeostasis by NPY is significantly involved in CR-mediated lifespan exten-sion, is triggered independently of classical longevity pathways, and is a potential therapeutic strategy to prevent obesity and frailty in elderly people.
